# Senile Systemic Amyloidosis Presenting as Hematuria: A Rare Presentation and Review of Literature

**DOI:** 10.1155/2020/5892707

**Published:** 2020-01-03

**Authors:** Thejus Jayakrishnan, Amir Kamran, Deep Shah, Aritra Guha, Muhammad Salman Faisal, Prerna Mewawalla

**Affiliations:** Division of Hematology Oncology, Department of Medicine, Allegheny Health Network, Pittsburgh, PA, USA

## Abstract

**Introduction:**

Senile systemic amyloidosis is a multisystem disease where wild-type insoluble transthyretin (ATTRwt) protein gets deposited in the tissues leading to organ dysfunction.

**Methodology:**

We present the case of a patient who presented with hematuria and bladder involvement by ATTRwt amyloidosis who ultimately died of multiorgan failure.

**Results:**

The patient was an 82-year-old white male with a history of ischemic cardiomyopathy (ejection fraction (EF): 20–25%), chronic atrial fibrillation, chronic kidney disease (CKD), and carpal tunnel syndrome who presented with acute hematuria, urinary retention, and progressive fatigue. He underwent cystoscopy and bladder biopsy which was positive on congo-red stain diagnostic for amyloidosis. Echocardiogram demonstrated worsening of EF to 10–15% and concentric left ventricle hypertrophy. MRI was not performed due to underlying CKD. His condition deteriorated during the hospital stay, and he developed cardiogenic shock and progressive liver dysfunction. Infectious workup was negative. Meanwhile, the biochemical investigations (serum protein electrophoresis, immunofixation, and urine kappa/lambda chains) ruled out plasma cell dyscrasias. Mass spectrometry analysis of the bladder biopsy specimen confirmed wild-type transthyretin (ATTRwt) amyloidosis consistent with senile systemic amyloidosis. Due to patients' extremely poor prognosis, his family wished to focus on patient's comfort-oriented measures only, and patient passed away shortly thereafter.

**Conclusion:**

Senile systemic amyloidosis can rarely present in an atypical fashion such as hematuria. The treatment options are limited in this disease process. Novel therapies are in the early phases of development. Concern also exists that in patients with multiple comorbidities, this entity is under recognized until the more advanced stages.

## 1. Introduction

Amyloidosis is a disease caused by the deposition of abnormally folded protein fibrils in various organs. Among the different subtypes, the aTTR-type amyloidosis (transthyretin) is caused by the deposition of transthyretin protein and may be wild-type (senile systemic amyloidosis) or with mutation (hereditary amyloidosis). Accumulation of transthyretin proteins in the heart leads to senile cardiac amyloidosis [1].

Although all patients with senile amyloidosis may have deposits in the heart, the factors that predispose certain individuals to manifest heart failure from cardiac amyloidosis are not well understood. Failure to recognize amyloidosis and tailor treatment may be responsible for variation in response to heart failure treatments, especially the heart failure with preserved ejection fraction phenotypes [2]. Targeted therapies for senile amyloidosis are also in its early stages. In this context, we present an interesting case of an elderly gentleman with senile amyloidosis of unknown duration who presented with hematuria and rapidly developed cardiogenic shock and ultimately passed away. Review of literature in the last five years (2013–2018) on amyloidosis and cardiac involvement is also been presented.

## 2. Case Report

The patient was an 82-year-old white male with a history of ischemic cardiomyopathy status postcardiac stenting, history of heart failure with reduced ejection fraction (HFrEF with ejection fraction (EF): 20–25%) with implantable cardiac defibrillator (ICD), chronic atrial fibrillation on dabigatran, obstructive sleep apnea (OSA), chronic kidney disease (CKD) stage III, carpal tunnel syndrome, and gout who presented with fatigue, acute hematuria, and urinary retention. He was a nonsmoker, did not endorse regular alcohol use, and denied any illicit drug use. His family history was noncontributory. His vitals at presentation were temperature 36.3°C, heart rate 80 beats per minute, blood pressure 100/70 mm Hg, and oxygen Saturation 93% on room air. Home medications included bumetanide 1 mg daily, metolazone 1.25 mg twice a week, carvedilol 12.5 mg daily, and dabigatran 75 mg twice a day. Complete blood count (CBC) was unremarkable, and basic metabolic panel (BMP) showed creatinine of 2.2 mg/dl (near baseline). NT-pro-BNP was elevated at 18,500 pg/ml (baseline 2000s). Troponin was elevated at 0.36 ng/ml.

Urology was consulted, and patient underwent cystoscopic evaluation for hematuria. Clot evacuation and fulguration of diffuse bladder lesions were performed. Bladder biopsy was also obtained, and subepithelial amyloid deposits were identified based on Congo-red stain.

In the following days, the patient developed intermittent episodes of atrial fibrillation with rapid ventricular rate, hypotension, liver dysfunction, and acute kidney injury secondary to acute tubular necrosis. Aspartate aminotransferase (AST) peaked at 3000 (U/L) and alanine aminotransaminase (ALT) at 2000 (U/L). Remaining liver function tests showed alkaline phosphatase 50 (U/L), bilirubin 3-4 mg/dl, hyperammonemia (72 mcmol/L) and elevated international normalized ration (INR) around 4. Computed tomography imaging of chest, abdomen, and pelvis was negative for any acute process but showed cardiomegaly that was seen in the plain chest radiography as well. Workup for any acute infectious or inflammatory processes (viral markers, acetaminophen levels, and autoimmune markers) for evaluation of acute liver dysfunction did not reveal any alternate diagnosis, thereby suggesting acute decompensation from shock liver.

**Figure 1:**
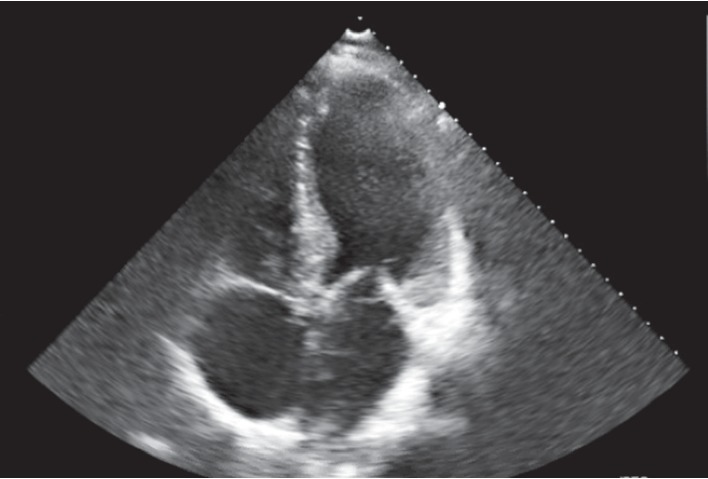
Echocardiogram showing normal biventricular dimensions with biatrial dilation with no significant valvular abnormalities.

Echocardiogram demonstrated worsening of ejection fraction (EF) to 10–15% with concentric left ventricle hypertrophy, impaired left ventricular diastolic dysfunction, thickened interventricular septum more than 12 mm, normal biventricular dimensions, biatrial dilation with no significant valvular abnormalities (findings compatible with cardiac amyloidosis) (Figure 1). Magnetic Resonance Imaging (MRI) was not performed due to underlying CKD. His condition continued to deteriorate, and he was transferred to the cardiac care unit for acute decompensated heart failure. He was started on milrinone infusion with transient improvement in his cardiac function and end organ perfusion.

He was evaluated for cardiac resynchronization therapy upgrade but deemed unstable from medical standpoint to undergo the procedure.

Hematology team was consulted and performed biochemical workup for amyloidosis. Serum protein electrophoresis (SPEP) did not show an M-spike. Serum-free light analysis (kappa/lambda ratio 2.9) and urine immunofixation studies were not significant ruling out evidence for plasma cell dyscrasias. Meanwhile, the mass spectrometry analysis of the bladder biopsy specimen confirmed wild-type transthyretin (ATTRwt) consistent with senile systemic amyloidosis. Immunohistochemical analysis was not performed as mass spectrometry has been demonstrated to be a well-validated technique to diagnose and classify amyloidosis [3, 4]. Genetic testing can help clarify if the disease was hereditary or not but was not performed in this case and remains a limitation.

Multiple specialties including internal medicine, hematology, cardiology, and critical care were involved in the patient care, but despite aggressive management, patient's condition gradually deteriorated. Due to the poor prognosis in the setting of multiple comorbidities and decompensated heart failure, the patients' family decided to stop further treatment and focus on patients comfort only which was facilitated by the palliative care team. The patient passed away shortly thereafter from cardiac arrest, and the diagnosis was acute on chronic cardiac failure from senile systemic amyloidosis.

## 3. Discussion

Amyloidosis is a disorder where there is damage and functional compromise of tissue secondary to extracellular deposition of fibrillary proteins [5]. There are several different ways of classifying amyloidosis. Based on the type of protein that is deposited, it may be classified into AL (amyloid light deposition of light chains in the setting of monoclonal plasma cell proliferation), AA (acute phase reactant serum amyloid A-derived amyloidosis in the setting of chronic inflammation), ATTRwt or senile systemic amyloidosis (wild-type transthyretin deposition), and ATTRv (mutated or hereditary type as a result of mutations in genes encoding normally soluble proteins including transthyretin protein) [6]. International Society of Amyloidosis meets every two years to provide updates on the disease. Recently, it was recommended to use hereditary amyloidosis uniformly instead of familial amyloidosis [7].

Although senile systemic amyloidosis or ATTwt may involve urinary bladder, presentation as hematuria leading to the diagnosis is not well described and requires high index of suspicion [8]. Atypical presentations do exist with occasional fatal outcomes during the index hospitalization. For instance, authors from Japan have described a case of alveolar hemorrhage and death secondarily to ATTRwt amyloid deposition in bronchial artery in a patient admitted with AHF (acute heart failure) [9]. This indicates the need for heightened awareness of this entity to facilitate early diagnosis.

As could be expected, the prevalence of ATTRwt increases with age. Among octogenarians, 25% have evidence of transthyretin (ATTR) deposits (both wild and hereditary types) in tissue on autopsy. Cardiac ATTR deposits have been identified in up to 5% of hypertrophic cardiomyopathy patients and 13% of heart failure with preserved ejection fraction (HFpEF) [10]. The median age of diagnosis of heart failure from senile amyloidosis is 75 years (range 59–90) with a median survival of 2.6 years after diagnosis [11]. Pertinent to our case is the finding that carpal tunnel syndrome may be a common initial presentation of patients with senile systemic amyloidosis presenting on an average of 5 years prior to diagnosis of amyloidosis [12]. In our patient, this duration was 7 years.

Studies have suggested ATTRwt as an under-diagnosed cause of HFpEF, and that efforts can be made to identify these patients early to tailor heart failure therapies as well as targeted therapies for amyloidosis [13]. In a study, among HFpEF patients aged ≥75 years, 32% had ATTRwt deposits in their heart versus 8% of patients <75 years of old. Notably, only 20% of these patients had a premortem diagnosis of amyloidosis [14]. While most cases of cardiac amyloidosis present as hypertrophic cardiomyopathy (HCM), cases where patients initially have dilated cardiomyopathy (DCM) have also been described [15]. Management of these patients hinges mainly on volume and arrhythmia management as these patients poorly tolerate neurohormonal antagonists generally used for heart failure [16]. Cases similar to our patient have been described where patients remain undiagnosed with cardiac amyloidosis for a long time despite having cardiomyopathy [17]. In our case, patient suffered from ischemic heart disease and may have been a reason why further evaluation that could have led to the diagnosis of cardiac amyloidosis was not performed. Patients with cardiac amyloidosis who present with heart failure (HF) suffer from high mortality of around 50%, with risk higher among those who develop cardiogenic shock (80%) [18]. Cases of patients with AL-type amyloidosis presenting with acute heart failure have also been described, and these patients have worse outcomes compared with ATTRwt probably from the aggressive nature of the disease process [18, 19].

From a population standpoint, studies have shown higher prevalence of HF secondary to nonischemic cardiomyopathy (NICM) as well as cardiac amyloidosis among African races. In a large volume retrospective study from London, cardiac amyloidosis (11%, all types) was the fourth most common cause of heart failure in Afro-Caribbean with a higher prevalence of NICM (27%) compared with Caucasians (19%). Also of interest, the majority of Afro-Caribbean patients with cardiac amyloidosis had ATTR V122I mutation portending worst prognosis among the subgroups [11].

With regard to biochemical markers, cardiac amyloidosis in patients with HF is associated with higher BNP [13]. NT-pro-BNP obtained in a stable hemodynamic state has been shown to be the only short term predictor of cardiogenic shock with a sensitivity of 92% and specificity of 81% [18].

While tissue biopsy demonstrating amyloid deposits is the gold standard for diagnosis cardiac amyloidosis, recent studies have reported high accuracy of cardiac MRI with T1 mapping and extracellular volume (ECV) estimation [20]. MRI is also valuable in distinguishing cardiac amyloidosis (CA), HCM, and hypertensive heart disease [21]. Bone scintigraphy has also been proposed as an alternative modality for noninvasive diagnosis of cardiac amyloidosis (excluding the AL-type amyloidosis) with high accuracy [13, 22]. Combination of characteristic findings on imaging (echo or MRI) and biopsy of noncardiac tissue such as urinary bladder as in our case is also a viable option.

The fact remains that diagnosis of cardiac amyloidosis requires high index of suspicion due to overlap with other common causes of cardiomyopathy. Significant number of patients with cardiac amyloidosis may remain asymptomatic for a long time until their comorbidities such as diabetes, hypertension, and chronic kidney disease independently impair cardiac function and accelerate ATTR deposition by promoting a prooxidative state [1]. The onus for early diagnosis of cardiac amyloidosis in these patients may be upon the PCPs or sometime the hematologists who may see them for systemic amyloidosis as in the current case. It has been proposed that patients who have risk factors such as male gender, age ≥65 yrs, heart failure symptoms, symmetric left ventricular (LV) hypertrophy, and moderately depressed or HFpEF should undergo screening for amyloidosis [17]. Machine learning models have also been investigated and may provide novel insights into early diagnosis of ATTRwt patients [23]. There are other clinical implications as well. For instance, not identifying patients with cardiac amyloidosis and thereby resulting in a heterogeneous study group with confounding effects have been a criticism for the major heart failure trials such as I-PRESERVE and TOPCAT [2].

Genetic testing is another area of interest in the paradigm of amyloidosis. World's most prevalent amyloidogenic mutation responsible for hereditary amyloidosis (ATTRv) involves valine to isoleucine amino acid substitution at position 122 (V122I) with a prevalence of 3.4% and clinical penetrance of around 10% in carriers [24]. While the utility of universal screening of patients or patients at high risk for heart failure may be questionable, there may be value in genetic analysis in those who established heart failure especially as the costs for genetic testing gets cheaper. Whether this has any predictive or prognostic value would only be established once this subset is adequately studied [24]. Physicians encountering amyloidosis patients are encouraged to be involved in the Transthyretin Amyloidosis Outcomes Survey (THAOS), an international, longitudinal, observational study designed to investigate the disease course and its management among symptomatic patients as well as their asymptomatic mutation-carrying family members [25].

**Figure 2:**
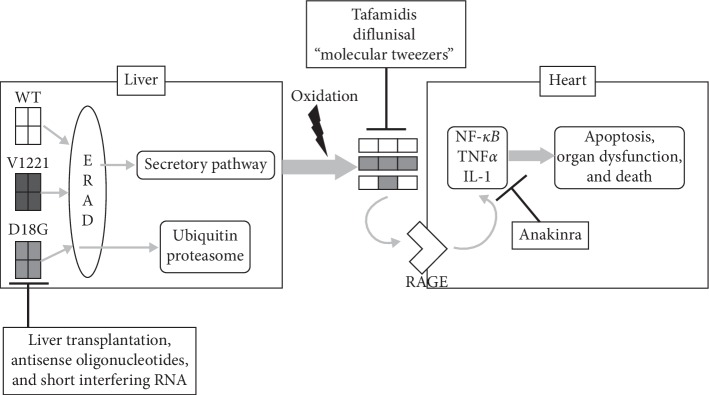
Diagram with proposed mechanism and the various therapeutic targets for treatment of senile amyloidosis [1] (reproduced from prepublished journal article that is available as an open access, free to reproduce format; refer to [1] for more details).

The treatment options for amyloidosis are evolving [26–28]. A traditional treatment option was orthotopic liver transplantation (OLT) that eliminates significant amount of ATTR from the blood and helps alter the course of the disease. Limitations of OLT including the need for careful patient selection, prolonged immunosupression in patients, and reports of disease progression has led to the development of new drugs [26]. These include drugs that stabilize the ATTR molecule, silence the production of ATTR or accelerate the degradation (Figure 2). Although thyroxine is a natural ligand for ATTR, it cannot be used in the therapeutic setting due to its hormonal properties. Two drugs that have been shown to stabilize the transthyretin molecule are diflunisal and tafamidis. Diflunisal is a nonsteroidal anti-inflammatory drug (NSAID) that has demonstrated improvement in patients with hereditary amyloidosis and shown to be tolerated by patients with cardiac failure despite its NSAID properties [29]. The therapeutic effectiveness remains to be entirely established [30, 31]. Tafamidis is another drug that has been shown to be effective. It functions by stabilizing the ATTR molecule and by virtue of not being an NSAID, it is likely safer for heart failure patients. In a multicenter international randomized clinical trial among patients with cardiac amyloidosis, tafamidis was shown to significantly reduce all-cause mortality (30% vs. 43%; hazard ratio (HR) 0.7; 95% confidence interval (CI), 0.51 to 0.96), cardiovascular-related hospitalizations (relative risk ratio, 0.7; 95% CI, 0.56 to 0.81) and significantly reduce the decline in functional capacity and quality of life compared with the placebo [31]. It was approved recently by United States Food and Drug Administration (US FDA) and European Medical Agency (EMA) for use as a breakthrough drug in cardiac amyloidosis [32, 33].

The ATTR silencers in the forms of ATTR siRNA (small interfering RNA) conjugated with molecules that can deliver it to hepatocytes are in phase II/III clinical trials [30]. In phase 2 trials, these were shown to cause 98.2% knockdown of serum ATTR, including both wild and mutant ATTR [34]. Novel therapies that prevent deposition of amyloid proteins or accelerate the degradation (Anakinra, an Il-1 type 1 receptor inhibitor, lysine-specific molecular tweezers, doxycycline and EGCG, a polyphenolic compound in green tea) have also shown promising outcomes *in vitro* and are being studied in early clinical trials [1].

## 4. Conclusion

Senile systemic amyloidosis or ATTRwt may rarely present in an atypical fashion such as hematuria and subsequent severe acute multiorgan dysfunction as in our case. Prognosis is poor in those cases due to limited treatment options. While the only definitive treatment for transthyretin amyloidosis is orthotopic liver transplant, as the source of the amyloidogenic protein is the liver, most patients are ineligible due to comorbidities. Novel therapies such as ATTR fibril stabilizing proteins and gene silencers are evolving. Concern exists that, in patients with multiple comorbidities, this entity is under recognized until the more advanced stages, especially in the face of an aging population.
